# The moderating role of aerobic exercise in the relationship between stress and cognitive functions

**DOI:** 10.1038/s41598-025-14202-w

**Published:** 2025-08-09

**Authors:** Mert Ayranci, Mehmet Kemal Aydin, Metin Kuş, Murat Sarikabak, Mehmet İsmail Tosun, Servet Ebrar Bayram, Mehmet Civan

**Affiliations:** 1 Faculty of Sport Sciences, Hitit University, Çorum, Turkey; 2 Center for Distance Education Application and Research, Hitit University, Çorum, Turkey; 3 Faculty of Sport Sciences, Bartın University, Bartın, Turkey

**Keywords:** Aerobic exercise, Cognitive functions, Stress, Sleep, Moderation analysis, Human behaviour, Psychology and behaviour

## Abstract

This study examines the effect of stress on cognitive failures and the potential moderating role of aerobic exercise. A total of 290 university students participated, and moderation analysis was conducted using Hayes’ PROCESS Model 1 (Version 4.2). Results showed that stress alone did not significantly predict cognitive failures. However, aerobic exercise appeared to play a potential moderating role in this relationship. Specifically, a significant association between stress and cognitive failures was observed among moderate- and high-intensity exercisers, while no such effect was found in low-intensity exercisers. Additionally, sleep duration was negatively associated with cognitive failures. These findings suggest that aerobic exercise may influence the relationship between stress and cognitive failures, although further investigation is needed to establish this effect more conclusively.

## Introduction

In modern life, individuals frequently experience high levels of stress due to academic, professional, and social obligations. While acute stress can enhance adaptive responses by improving focus and reaction times, chronic stress has been linked to adverse effects on both physical and cognitive functions^1–3^. Research indicates that prolonged exposure to stress impairs memory, attention, and executive functions, increasing the likelihood of cognitive failures^4,5^.

Cognitive failure refers to a pattern of forgetfulness, inattentiveness, and errors in information processing^6^. The detrimental impact of stress on cognitive functions is primarily attributed to its effects on the hippocampus and prefrontal cortex. Chronic stress has been shown to reduce hippocampal plasticity, thereby impairing learning and memory processes, while simultaneously weakening prefrontal cortex functions, which are essential for executive control and decision-making^7^.

A growing body of research highlights the regulatory role of physical exercise in mitigating the negative effects of stress on cognitive functions. Regular aerobic exercise has been found to lower cortisol levels, enhance neural plasticity, and improve overall cognitive performance^8,9^. Studies suggest that individuals with higher physical activity levels may be more resilient to the cognitive impairments associated with stress^10,11^. However, further evidence is needed to determine the extent to which exercise moderates the relationship between stress and cognitive failure.

Another critical factor influencing cognitive performance is sleep. Insufficient sleep has been linked to deficits in attention, memory, and executive functions while also exacerbating stress levels. Given its potential bidirectional relationship with both stress and cognition, sleep duration and quality must be considered when examining the moderating role of aerobic exercise^12,13^. Additionally, given the well-established relationship between sleep quality and cognitive performance, controlling for sleep duration was considered essential to isolate the specific effects of stress and aerobic exercise on cognitive failures.

Given this context, the present study aims to investigate the impact of stress on cognitive functions and examine whether aerobic exercise moderates this relationship. Additionally, the role of sleep duration and quality in cognitive processes was assessed. In this context, the following hypotheses were tested:

### H_1_

When sleep duration is controlled, stress is positively and significantly associated with cognitive failures, indicating that higher stress levels lead to greater cognitive impairments.

### H_2_

It was hypothesized that aerobic exercise would moderate the relationship between stress and cognitive failures, such that the positive association between stress and cognitive failures would be weaker among individuals with higher levels of aerobic exercise.

## Methods

**Fig. 1:**
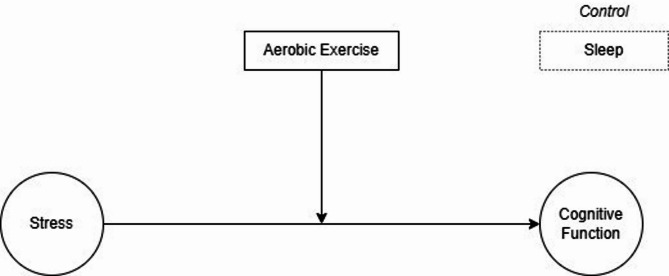
The hypothesized moderation model.

The present study employed a correlational research design to investigate the impact of stress on cognitive functions and the moderating role of aerobic exercise in this relationship. Correlational research is a quantitative methodology that examines natural associations between variables and determines their direction and strength^14^. Additionally, the study utilized Andrew F. Hayes’ Moderation Model 1 to assess how aerobic exercise levels influence the relationship between stress and cognitive functions^15^. In this model, stress was designated as the independent variable, cognitive function as the dependent variable, aerobic exercise level as the moderator, and sleep duration as a control variable. Figure 1 illustrates the hypothesized research model.

### Participants

The study sample consisted of 158 male (54.5%) and 132 female (45.5%) undergraduate students enrolled in bachelor’s degree programs at a state university in the Central Black Sea Region of Turkey. Based on the average scores obtained from the IPAQ-SF, participants were categorized into three groups: 74 (26%) engaged in low-intensity aerobic exercise, 95 (32.8%) engaged in moderate-intensity aerobic exercise, and 121 (41.7%) engaged in high-intensity aerobic exercise.

Prior to data collection, an ethical approval letter was retrieved from the Non-Interventional Research Ethics Committee of Hitit University. Participants were informed about the study’s purpose and procedures, provided informed consent, and all data were processed anonymously in compliance with confidentiality principles. The study adhered to the ethical guidelines outlined in the Declaration of Helsinki.

To determine the appropriate sample size, a power analysis was conducted using G*Power 3.1 software^16^. Based on a 95% confidence level, 90% statistical power, and a medium-to-high effect size (f^2^ = 0.20), a minimum of 265 participants was deemed sufficient for the study. During the data analysis process, outlier detection procedures were implemented, leading to the exclusion of data from eight participants who exhibited statistically significant deviations. Consequently, the final sample size for the study was 290.

### Data collection tools

#### Cognitive functions scale

This scale, originally developed by Broadbent et al. (1982), was adapted into Turkish by Ekici et al. (2016) and is locally known as the Bilişsel Durum Ölçeği (BDÖ)^6,17^. It consists of 25 items, each rated on a 5-point Likert scale ranging from 0 (Never) to 4 (Very often), yielding a total score range of 0 to 100, a higher score on the scale indicates a greater frequency of cognitive failures, reflecting diminished cognitive functioning in daily life. Example items include: “Do you forget people’s names?” and “Do you find yourself dropping things?”. The Cronbach’s alpha for this study was calculated as 0.91.

#### International physical activity questionnaire-short form (IPAQ-SF)

This scale developed by Michael Booth in 1996 was adapted into Turkish in 2005. The scale includes questions about PA performed for at least 10 min in the last 7 days. One MET-minute is calculated by multiplying the minutes of activity by the MET score. A person at rest consumes 3.5 ml of oxygen (1 MET) per kg per minute. According to IPAQ data, Heavy Physical Activity =  8.0 METs, Moderate Physical Activity =  4.0 METs, Normal Walking (4–5 km/h) =  3.3 METs. In this study, aerobic exercise intensity was computed exclusively using activity types classified as aerobic within the IPAQ-SF structure. Specifically, MET values were calculated based on participants’ self-reported duration and frequency of walking (3.3 METs), moderate physical activity (4.0 METs), and vigorous physical activity (8.0 METs), such as aerobic workouts, cycling, and fast-paced sports. Other activity domains—such as occupational or household physical tasks—were excluded from MET calculations. This ensured that exercise intensity classification was based solely on aerobic activity patterns in line with IPAQ guidelines. Croanbach’s alpha coefficient for this study was calculated as 0.87.

#### Richard campbell sleep questionnaire (RCSQ)

The Sleep Quality Scale was developed by R. Campbell, and its Turkish adaptation was carried out by Karaman-Özlü and Özer^18,19^. The 6-item scale is designed so that higher scores indicate better sleep quality. The Cronbach’s alpha coefficient for the scale was found to be 0.77, demonstrating acceptable internal consistency. Sleep duration was operationalized using the relevant item from the RCSQ, which asked participants to report the average number of hours slept per night.

#### Perceived stress scale short form

The Perceived Stress Scale was developed by Schäfer et al. (2023) and was translated into Turkish by Kocapınar and Ekşi^20,21^. A higher score on the scale indicates a higher level of perceived stress. The scale includes two negatively worded items, and the Cronbach’s alpha coefficient was calculated as 0.74, indicating acceptable internal consistency.

### Data analysis

The data analysis was conducted using IBM SPSS Statistics 26.0 and Hayes’ PROCESS Macro (Version 4.2). To assess the distribution characteristics of the data, the Kolmogorov-Simirnov test was performed. The results indicated that the main variables met the assumption of normality (*p* > 0.05), thereby justifying the use of parametric analyses. Prior to the main analyses, outlier detection was conducted using a Z-score threshold of ±  3.29. Based on this conservative criterion, eight participants were identified as outliers and excluded from the dataset. Subsequently, Pearson correlation analysis was conducted to examine the bivariate relationships among variables. To test the impact of perceived stress on cognitive failures and the moderating role of aerobic exercise intensity in this relationship, Moderation Model 1 from Hayes’ PROCESS Macro was employed. In this model, perceived stress was entered as the independent variable, cognitive failures as the dependent variable, aerobic exercise intensity as the moderator, and sleep duration as a covariate. The significance of the interaction term (X *×* W) was tested using 5000 bootstrap resamples to generate bias-corrected confidence intervals. Simple slopes analysis was conducted to probe significant interactions, and line graphs were generated to visualize the moderation effect. For all analyses, the significance level was set at *p* < 0.05.

## Results

Table 1 presents the moderation analysis results examining the relationship between stress and cognitive failures, with aerobic exercise intensity as a moderating variable and sleep duration as a control variable.

**Table 1:** The effect of stress on cognitive failures: Moderation analysis results.

Variables	B	SE	*t*	*p*	95% CI
Constant	2.451	0.574	4.269	0.001	[1.321, 3.581]
Stress (X)	− 0.105	0.177	− 0.591	0.556	[− 0.455, 0.245]
AE intensity (W)	− 0.411	0.237	− 1.732	0.084	[− 0.879, 0.056]
Stress X AE intensity	0.130	0.076	1.700	0.090	[− 0.021, 0.281]
Sleep (control)	− 0.043	0.020	− 2.158	0.032	[− 0.084, − 0.003]

According to the results of the regression analysis, the model’s explanatory power for the dependent variable (cognitive function) was calculated as R^2^ =  0.0528 (5.28%), indicating that the model is statistically significant overall (*F*(4, 285) = 3.97, *p* = 0.0038). However, the direct effect of stress on cognitive function was not statistically significant (B = − 0.105, *p* = 0.556), suggesting that stress alone does not significantly affect cognitive function. The direct effect of aerobic exercise intensity, included as a moderator variable, was not statistically significant but was close to significance (B = − 0.411, *p* =  0.084), implying that aerobic exercise might have a potential influence on cognitive function. The interaction term between stress and aerobic exercise (B = 0.130, *p* =  0.090) did not reach conventional levels of statistical significance. Therefore, this result should be interpreted with caution, though it may suggest a potential moderating effect that warrants further investigation.. Regarding the control variable (sleep duration), results showed a significant negative effect on cognitive function (B =− 0.043, *p* = 0.032), indicating that as sleep duration increases, cognitive function scores decrease (interpreted as improved cognitive performance in the study). These findings suggest that the effect of stress on cognitive function may vary depending on aerobic exercise levels and that this relationship may differ under specific conditions.

Table 2 presents the detailed moderation effects of exercise level on the relationship between stress and cognitive function, highlighting how varying exercise levels influence this association. The moderation analysis findings indicate that exercise level significantly alters the effect of stress on cognitive function. At a low level of exercise, the effect of stress on cognitive function was not statistically significant (B = 0.0255, *p* = 0.8163), suggesting that stress does not influence cognitive function in individuals with low exercise levels. However, among individuals engaging in moderate levels of exercise, the effect of stress on cognitive function became significant (B = 0.1560, *p* = 0.0173). This finding suggests that moderate exercise may enhance the effect of stress on cognitive function, potentially leading to increased cognitive impairment. When exercise levels are high, the effect of stress on cognitive function becomes strongest and is highly statistically significant (B = 0.2866, *p* = 0.0017). These results suggest that increasing exercise levels strengthens the positive relationship between stress and cognitive function scores (interpreted as cognitive impairment in this study). This highlights the moderating role of exercise in the relationship between stress and cognitive function, suggesting that exercise may influence how stress affects cognitive performance.

**Table 2:** Conditional effects of moderation analysis (effect of stress by aerobic exercise intensity).

AE Intensity	Stress (B)	SE	*t*	*p*	%95 CI
Low	0.0255	0.1098	0.2325	0.081	[− 0.190, 0.241]
Moderate	0.1560	0.0652	2.3951	0.017	[0.027, 0.284]
High	0.2866	0.0907	3.1612	0.001	[0.108, 0.465]

**Fig. 2:**
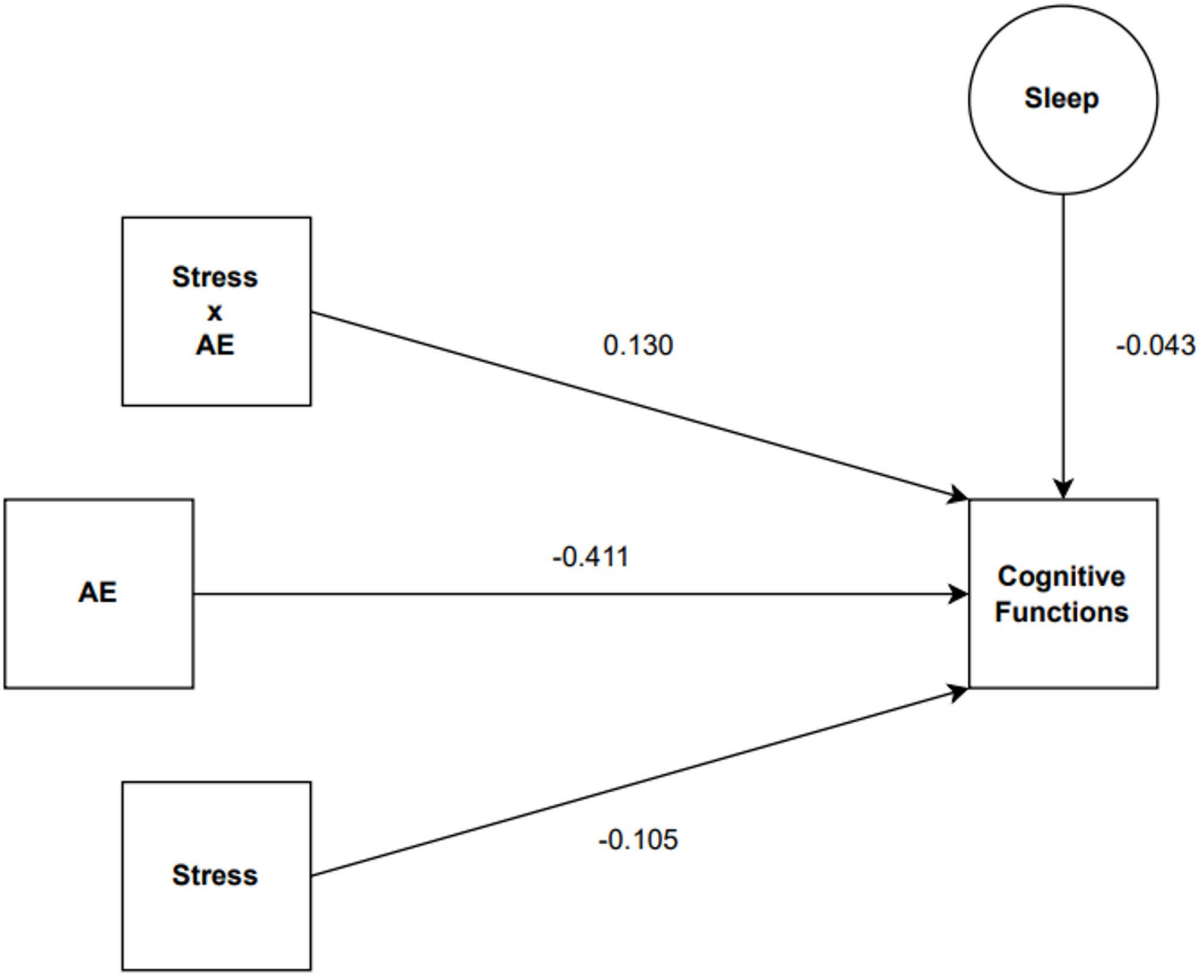
Structural model between stress, aerobic exercise, sleep and cognitive functioning.

The model is presented in Fig. 2. This hypothetical moderation model assumes that the effect of stress on cognitive failure is moderated by exercise level. In the model, stress is designated as the independent variable, cognitive failure as the dependent variable, exercise level as the moderator variable, and sleep duration as the control variable. The structural framework has been designed in accordance with Hayes’ Moderation Model 1.

**Fig. 3:**
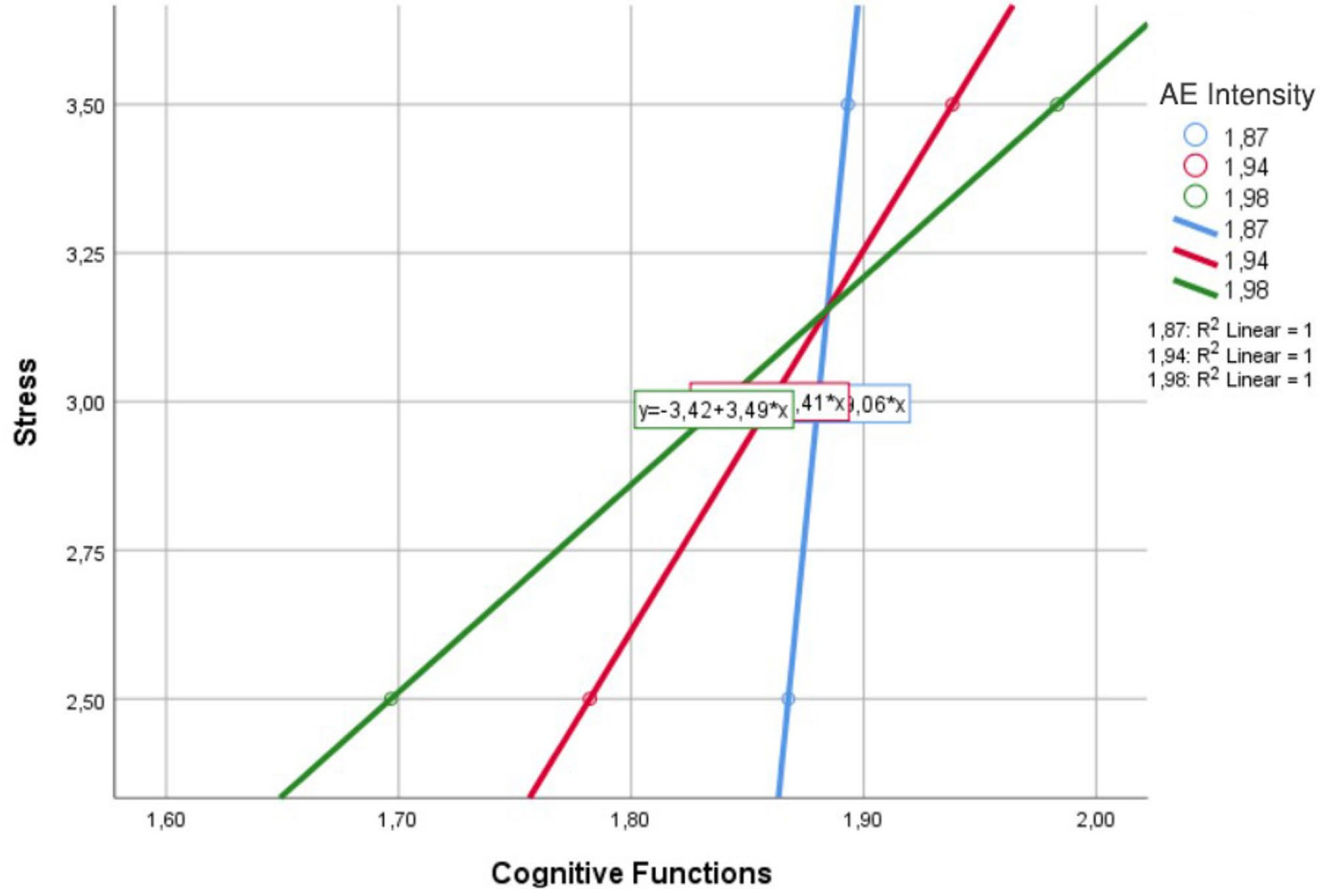
Interaction model between stress, aerobic exercise intensity and cognitive functioning.

Figure 3 illustrates the changes in cognitive functions as stress levels increase and the moderating effect of aerobic exercise (AE) intensity on this relationship. The findings indicate that at low exercise intensity, cognitive function scores increase as stress levels rise. However, at moderate and high exercise intensities, this relationship reverses. This suggests that moderate and high-intensity exercise may alter the effect of stress on cognitive functions or mitigate its negative impact.

## Discussion and Conclusion

This study aimed to examine the effect of stress on cognitive failure and the moderating role of aerobic exercise in this relationship. While the direction of the interaction contradicted our original hypothesis (H2), its marginal significance also suggests caution in interpreting this effect. Given that the interaction term narrowly missed conventional significance (p = 0.090), this result should be interpreted with caution. Although suggestive, it does not provide definitive support for moderation. Furthermore, the modest explanatory power of the model (R^2^ = 0.0528) indicates that other unmeasured factors such as individual mood, recovery habits, or exercise frequency may influence cognitive outcomes and should be explored in future research. The findings indicate that stress alone does not have a significant effect on cognitive failure; however, exercise level significantly alters this relationship. While the effect of stress on cognitive failure was not statistically significant in individuals with low exercise levels, a significant relationship was observed among individuals engaging in moderate and high levels of aerobic exercise. These results tentatively suggest that aerobic exercise may moderate the relationship between stress and cognitive failure, although the direction of this effect was contrary to expectations and should be interpreted with caution. Additionally, sleep duration was found to be negatively associated with cognitive failure, a finding that aligns with previous research demonstrating the adverse effects of sleep deprivation on cognitive functions^22,23^.

The results of the regression analysis revealed that stress alone does not have a significant direct relationship with cognitive failure. However, the existing literature includes numerous studies indicating that stress can negatively impact cognitive functions^3,24^. Specifically, both acute and chronic stress have been reported to affect brain regions critical for cognitive functions, such as the prefrontal cortex and hippocampus, leading to impairments in working memory, executive functions, and attention processes^25^. However, some studies suggest that stress may enhance attention processes in the short term, indicating that the relationship between stress and cognition may vary depending on contextual factors^26^. In this context, the non-significant direct effect of stress in the present study suggests that its impact on cognitive functions may vary depending on individual lifestyle factors and environmental conditions.

One of the most significant findings of our study is that exercise level moderates the relationship between stress and cognitive failure. Contrary to our initial hypothesis (H2), the effect of stress on cognitive failure was not significant among individuals with low levels of aerobic exercise, yet it was positively significant at moderate and high levels. This finding suggests that, under certain conditions, higher exercise intensity may amplify the impact of stress on cognitive performance rather than mitigate it.

A potential explanation of this result involves the acute physiological effects of intense physical activity, such as short-term elevations in cortisol levels, which may temporarily impair cognitive processing^8^. Furthermore, mental fatigue or overtraining could also contribute to this pattern. While previous research has highlighted the protective effects of aerobic exercise on cognition through mechanisms like improved neural plasticity and BDNF regulation^9,27–31^. Our results suggest a more complex relationship. In this study, exercise levels were determined based on self-reported aerobic activities, minimizing the potential confounding effects of non-aerobic physical tasks commonly included in general physical activity questionnaires.

The moderating effect of exercise may also be linked to individual coping mechanisms for stress. Research has shown that regular physical activity reduces anxiety and depression symptoms, enhances stress tolerance, and supports cognitive flexibility^32–34^. These findings may explain why the effect of stress on cognition became significant among individuals with moderate and high levels of aerobic exercise in the present study. Although prior literature suggests that regular exercise may improve emotional regulation, our findings indicate that, in certain contexts, higher exercise intensity may correspond with increased cognitive failures under stress. This unexpected pattern warrants further investigation.In our study, sleep duration was found to be negatively associated with cognitive failure, indicating that an increase in sleep duration leads to a decrease in cognitive failure levels. This finding is consistent with previous research and aligns with studies demonstrating the negative effects of insufficient sleep on executive functions, attention processes, and memory consolidation^35–37^. Additionally, sleep deprivation can heighten the stress response, leading to an increase in cortisol levels, which may further weaken cognitive performance^3,38^. Previous studies have shown that exercise can enhance sleep quality, which may indirectly have a positive effect on cognitive processes^39^. Although the interaction between exercise and sleep was not directly tested in the present study, future research is recommended to explore this relationship in greater detail.

The findings of this study indicate that the effect of stress on cognitive failure is significantly moderated by aerobic exercise levels. Among individuals engaging in moderate and high levels of aerobic exercise, the effect of stress on cognition became statistically significant, whereas no significant relationship was observed in those with low exercise levels. Additionally, sleep duration was found to have a negative effect on cognitive failure, suggesting that longer sleep duration is associated with lower cognitive failure levels. These findings provide preliminary insights into the complex interplay between stress, aerobic exercise, and sleep, suggesting that these lifestyle factors may influence cognitive functioning in subtle and multifaceted ways.

## Limitations and Generalizability of findings

This study is significant as one of the few investigations exploring how aerobic exercise moderates the relationship between stress and cognitive failure, highlighting the potential role of physical activity in protecting cognitive functioning. However, several limitations should be acknowledged. Due to the cross-sectional nature of the design, no causal inferences can be drawn. Additionally, self-report instruments were used for key variables (e.g., physical activity, cognitive failures), which may introduce bias and reduce measurement precision.

Furthermore, the sample consisted solely of university students with a relatively narrow age range, which may limit generalizability to other age groups. Although gender was collected and reported as a demographic characteristic, it was not included as a covariate in the analyses. This choice was made to maintain parsimony and statistical power in the regression model, but future research should examine the potential moderating or confounding roles of demographic variables such as gender, age, mood, and exercise frequency.

For future research, experimental designs are recommended to explore causal effects, and longitudinal approaches could better capture the long-term influence of aerobic exercise on cognition. Additionally, comparisons between different types of exercise (e.g., aerobic vs. anaerobic) and analytical techniques like structural equation modeling may help to elucidate complex interactions between sleep, stress, and cognitive performance. Lastly, future studies should aim to identify the optimal type, intensity, and duration of physical activity that provides the greatest cognitive benefits.

## Data Availability

The data supporting the findings of this study are available on Figshare and can be accessed via DOI: [10.6084/m9.figshare.28629260]. The corresponding author MA can be contacted for data availability.
